# *N*-{2-[2-(2,6-Dichloro-3,5-dimethoxy­phen­yl)ethen­yl]phen­yl}acetamide

**DOI:** 10.1107/S1600536809003250

**Published:** 2009-01-31

**Authors:** Kartini Ahmad, Khalijah Awang, Noel F. Thomas, Jean-Frederic Faizal Weber, Seik Weng Ng

**Affiliations:** a Department of Chemistry, University of Malaya, 50603 Kuala Lumpur, Malaysia; b Faculty of Pharmacy, Universiti Teknologi MARA, 40450 Shah Alam, Selangor Darul Ehsan, Malaysia

## Abstract

The C=C double bond in the title substituted stilbene, C_18_H_17_Cl_2_NO_3_, has a *trans* arrangement of the aryl substit­uents. The aromatic ring of the 2-acetyl­amino­phenyl substit­uent is twisted by 39.9 (3)° with respect to the central C—C=C—C unit and that of the 2,6-dichloro-3,5-dimethoxy­phenyl substitutent is twisted by 42.7 (3)°.

## Related literature

The compound was synthesized by a ferric chloride-promoted highly atropodiastereoselective cascade reaction, a reaction that illustrates the utility of radical cations in indolostilbene synthesis; see: Ahmad *et al.* (2009 ▶).
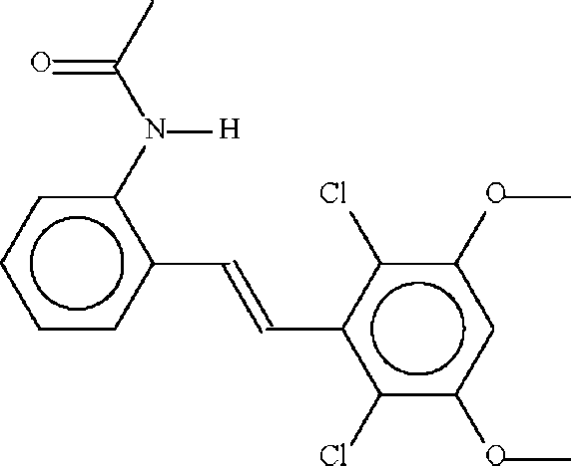


## Experimental

### 

#### Crystal data

C_18_H_17_Cl_2_NO_3_
*M* *_r_* = 366.23
Triclinic, 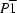

*a* = 7.5646 (3) Å
*b* = 9.1485 (3) Å
*c* = 12.2969 (5) Å
α = 78.561 (2)°
β = 77.716 (2)°
γ = 85.969 (3)°
*V* = 814.65 (5) Å^3^
*Z* = 2
Mo *K*α radiation
μ = 0.42 mm^−1^
*T* = 100 (2) K
0.30 × 0.03 × 0.03 mm

#### Data collection

Bruker SMART APEX diffractometer
Absorption correction: multi-scan (*SADABS*; Sheldrick, 1996 ▶) *T*_min_ = 0.886, *T*_max_ = 0.988
6677 measured reflections
3657 independent reflections
2490 reflections with *I* > 2σ(*I*)
*R*_int_ = 0.048

#### Refinement

*R*[*F* ^2^ > 2σ(*F* ^2^)] = 0.048
*wR*(*F* ^2^) = 0.117
*S* = 0.99
3657 reflections
220 parameters
H-atom parameters constrained
Δρ_max_ = 0.40 e Å^−3^
Δρ_min_ = −0.37 e Å^−3^

### 

Data collection: *APEX2* (Bruker, 2007 ▶); cell refinement: *SAINT* (Bruker, 2007 ▶); data reduction: *SAINT*; program(s) used to solve structure: *SHELXS97* (Sheldrick, 2008 ▶); program(s) used to refine structure: *SHELXL97* (Sheldrick, 2008 ▶); molecular graphics: *X-SEED* (Barbour, 2001 ▶); software used to prepare material for publication: *publCIF* (Westrip, 2009 ▶).

## Supplementary Material

Crystal structure: contains datablocks global, I. DOI: 10.1107/S1600536809003250/tk2361sup1.cif

Structure factors: contains datablocks I. DOI: 10.1107/S1600536809003250/tk2361Isup2.hkl

Additional supplementary materials:  crystallographic information; 3D view; checkCIF report

## Figures and Tables

**Fig. 1.**
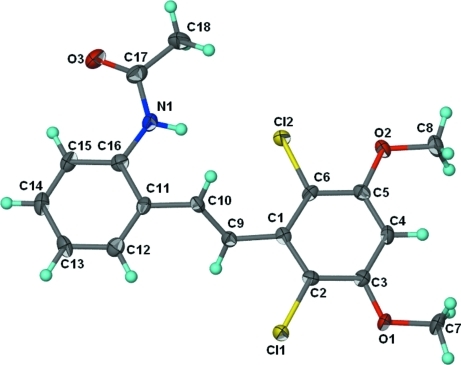
Thermal ellipsoid plot (Barbour, 2001) of C18H17Cl2NO3 at the 70% probability level. H atoms are drawn as spheres of arbitrary radius.

## supplementary crystallographic information

### Comment

The compound was synthesized by a ferric-chloride promoted, highly atropodiastereoselective cascade reaction, a reaction that illustrates the utility of radical cations in indolostilbene synthesis. The description of the synthesis is given in a recent study (Ahmad *et al.*, 2009).

### Experimental

The synthesis is described in an earlier report (Ahmad *et al.*, 2009).

### Refinement

Carbon-bound H atoms were placed in calculated positions (C—H 0.95–0.98 Å) and were included in the refinement in the riding model approximation, with *U*(H) set to 1.2–1.5*U*(C). The nitrogen-bound H-atom was similarly treated (N—H 0.88 Å, *U*(H) = 1.2*U*(N)).

### Crystal data

**Table:**

C_18_H_17_Cl_2_NO_3_	*Z* = 2
*M**_r_* = 366.23	*F*(000) = 380
Triclinic, *P*1	*D*_x_ = 1.493 Mg m^−^^3^
Hall symbol: -P 1	Mo *K*α radiation, λ = 0.71073 Å
*a* = 7.5646 (3) Å	Cell parameters from 1123 reflections
*b* = 9.1485 (3) Å	θ = 2.6–26.5°
*c* = 12.2969 (5) Å	µ = 0.42 mm^−^^1^
α = 78.561 (2)°	*T* = 100 K
β = 77.716 (2)°	Prism, colourless
γ = 85.969 (3)°	0.30 × 0.03 × 0.03 mm
*V* = 814.65 (5) Å^3^	

### Data collection

**Table:**

Bruker SMART APEX diffractometer	3657 independent reflections
Radiation source: fine-focus sealed tube	2490 reflections with *I* > 2σ(*I*)
graphite	*R*_int_ = 0.048
ω scans	θ_max_ = 27.5°, θ_min_ = 2.3°
Absorption correction: multi-scan (*SADABS*; Sheldrick, 1996)	*h* = −9→9
*T*_min_ = 0.886, *T*_max_ = 0.988	*k* = −11→11
6677 measured reflections	*l* = −15→15

### Refinement

**Table:**

Refinement on *F*^2^	Primary atom site location: structure-invariant direct methods
Least-squares matrix: full	Secondary atom site location: difference Fourier map
*R*[*F*^2^ > 2σ(*F*^2^)] = 0.048	Hydrogen site location: inferred from neighbouring sites
*wR*(*F*^2^) = 0.117	H-atom parameters constrained
*S* = 0.99	*w* = 1/[σ^2^(*F*_o_^2^) + (0.046*P*)^2^] where *P* = (*F*_o_^2^ + 2*F*_c_^2^)/3
3657 reflections	(Δ/σ)_max_ = 0.001
220 parameters	Δρ_max_ = 0.40 e Å^−^^3^
0 restraints	Δρ_min_ = −0.37 e Å^−^^3^

### Fractional atomic coordinates and isotropic or equivalent isotropic displacement parameters (Å^2^)

**Table:**

	*x*	*y*	*z*	*U*_iso_*/*U*_eq_	
Cl1	0.21992 (9)	0.40664 (7)	0.21221 (5)	0.02175 (18)	
Cl2	0.28411 (9)	0.66314 (7)	0.56854 (5)	0.01944 (18)	
O1	0.3311 (3)	0.68767 (19)	0.08280 (14)	0.0248 (5)	
O2	0.4014 (2)	0.90427 (18)	0.39962 (15)	0.0203 (4)	
O3	0.1911 (3)	0.2547 (2)	0.96831 (17)	0.0353 (5)	
N1	0.1666 (3)	0.3365 (2)	0.78526 (18)	0.0198 (5)	
H1	0.1734	0.4173	0.7322	0.024*	
C1	0.2581 (3)	0.5380 (3)	0.3861 (2)	0.0141 (5)	
C2	0.2725 (3)	0.5540 (3)	0.2691 (2)	0.0162 (6)	
C3	0.3281 (4)	0.6856 (3)	0.1940 (2)	0.0171 (6)	
C4	0.3763 (4)	0.8042 (3)	0.2354 (2)	0.0183 (6)	
H4	0.4190	0.8928	0.1845	0.022*	
C5	0.3624 (3)	0.7936 (3)	0.3506 (2)	0.0158 (6)	
C6	0.3045 (3)	0.6611 (3)	0.4245 (2)	0.0149 (5)	
C7	0.3852 (4)	0.8217 (3)	0.0026 (2)	0.0285 (7)	
H7A	0.3723	0.8102	−0.0729	0.043*	
H7B	0.5119	0.8405	0.0009	0.043*	
H7C	0.3085	0.9060	0.0250	0.043*	
C8	0.4638 (4)	1.0415 (3)	0.3273 (2)	0.0232 (6)	
H8A	0.4852	1.1118	0.3735	0.035*	
H8B	0.3721	1.0843	0.2836	0.035*	
H8C	0.5768	1.0225	0.2753	0.035*	
C9	0.1833 (3)	0.4005 (3)	0.4615 (2)	0.0154 (6)	
H9	0.0883	0.3559	0.4413	0.019*	
C10	0.2381 (3)	0.3337 (3)	0.5558 (2)	0.0144 (5)	
H10	0.3401	0.3734	0.5723	0.017*	
C11	0.1548 (3)	0.2039 (3)	0.6366 (2)	0.0159 (6)	
C12	0.1058 (4)	0.0794 (3)	0.6023 (2)	0.0193 (6)	
H12	0.1223	0.0778	0.5238	0.023*	
C13	0.0330 (4)	−0.0426 (3)	0.6812 (2)	0.0207 (6)	
H13	−0.0016	−0.1264	0.6565	0.025*	
C14	0.0106 (4)	−0.0427 (3)	0.7955 (2)	0.0222 (6)	
H14	−0.0367	−0.1275	0.8492	0.027*	
C15	0.0568 (4)	0.0805 (3)	0.8323 (2)	0.0203 (6)	
H15	0.0404	0.0803	0.9111	0.024*	
C16	0.1273 (4)	0.2042 (3)	0.7536 (2)	0.0178 (6)	
C17	0.1954 (4)	0.3555 (3)	0.8874 (2)	0.0234 (6)	
C18	0.2350 (4)	0.5142 (3)	0.8907 (2)	0.0289 (7)	
H18A	0.2924	0.5147	0.9549	0.043*	
H18B	0.1216	0.5736	0.8993	0.043*	
H18C	0.3165	0.5572	0.8201	0.043*	

### Atomic displacement parameters (Å^2^)

**Table:**

	*U*^11^	*U*^22^	*U*^33^	*U*^12^	*U*^13^	*U*^23^
Cl1	0.0352 (4)	0.0150 (3)	0.0164 (3)	−0.0059 (3)	−0.0067 (3)	−0.0029 (3)
Cl2	0.0257 (4)	0.0188 (3)	0.0144 (3)	−0.0049 (3)	−0.0029 (3)	−0.0043 (3)
O1	0.0446 (13)	0.0180 (10)	0.0117 (9)	−0.0104 (9)	−0.0079 (9)	0.0034 (8)
O2	0.0262 (11)	0.0149 (9)	0.0198 (10)	−0.0069 (8)	−0.0029 (8)	−0.0033 (8)
O3	0.0505 (14)	0.0387 (12)	0.0172 (11)	−0.0163 (11)	−0.0106 (10)	0.0027 (10)
N1	0.0279 (13)	0.0169 (11)	0.0133 (11)	−0.0032 (10)	−0.0023 (10)	−0.0004 (9)
C1	0.0100 (12)	0.0137 (12)	0.0179 (13)	0.0009 (9)	−0.0022 (10)	−0.0028 (10)
C2	0.0182 (14)	0.0156 (12)	0.0161 (13)	−0.0017 (10)	−0.0053 (11)	−0.0039 (11)
C3	0.0192 (14)	0.0166 (13)	0.0153 (13)	−0.0008 (11)	−0.0042 (11)	−0.0015 (11)
C4	0.0227 (15)	0.0133 (12)	0.0166 (14)	−0.0038 (11)	−0.0019 (11)	0.0016 (11)
C5	0.0157 (13)	0.0139 (12)	0.0192 (14)	0.0007 (10)	−0.0047 (11)	−0.0056 (11)
C6	0.0151 (13)	0.0183 (13)	0.0103 (12)	0.0009 (10)	−0.0016 (10)	−0.0019 (10)
C7	0.0440 (19)	0.0234 (15)	0.0151 (14)	−0.0083 (14)	−0.0060 (13)	0.0057 (12)
C8	0.0253 (16)	0.0149 (13)	0.0282 (16)	−0.0048 (11)	−0.0065 (13)	0.0012 (12)
C9	0.0195 (14)	0.0121 (12)	0.0149 (13)	−0.0008 (10)	−0.0027 (11)	−0.0037 (10)
C10	0.0153 (13)	0.0119 (12)	0.0158 (13)	−0.0029 (10)	−0.0005 (11)	−0.0038 (10)
C11	0.0165 (14)	0.0148 (13)	0.0150 (13)	0.0008 (10)	−0.0046 (11)	0.0016 (11)
C12	0.0195 (14)	0.0192 (13)	0.0183 (14)	0.0020 (11)	−0.0042 (11)	−0.0020 (11)
C13	0.0213 (15)	0.0108 (12)	0.0285 (16)	−0.0014 (11)	−0.0034 (12)	−0.0020 (11)
C14	0.0211 (15)	0.0190 (14)	0.0223 (15)	−0.0022 (11)	−0.0023 (12)	0.0046 (12)
C15	0.0213 (15)	0.0217 (14)	0.0147 (13)	0.0006 (11)	−0.0008 (11)	0.0008 (11)
C16	0.0182 (14)	0.0175 (13)	0.0178 (14)	0.0016 (11)	−0.0043 (11)	−0.0040 (11)
C17	0.0220 (15)	0.0321 (16)	0.0155 (14)	−0.0029 (13)	−0.0018 (12)	−0.0046 (13)
C18	0.0397 (19)	0.0290 (16)	0.0211 (15)	−0.0074 (14)	−0.0058 (14)	−0.0105 (13)

### Geometric parameters (Å, °)

**Table:**

Cl1—C2	1.742 (2)	C8—H8A	0.9800
Cl2—C6	1.748 (2)	C8—H8B	0.9800
O1—C3	1.359 (3)	C8—H8C	0.9800
O1—C7	1.437 (3)	C9—C10	1.333 (4)
O2—C5	1.355 (3)	C9—H9	0.9500
O2—C8	1.433 (3)	C10—C11	1.471 (3)
O3—C17	1.211 (3)	C10—H10	0.9500
N1—C17	1.365 (3)	C11—C12	1.389 (3)
N1—C16	1.411 (3)	C11—C16	1.410 (3)
N1—H1	0.8800	C12—C13	1.386 (4)
C1—C6	1.397 (3)	C12—H12	0.9500
C1—C2	1.399 (3)	C13—C14	1.379 (4)
C1—C9	1.476 (3)	C13—H13	0.9500
C2—C3	1.395 (3)	C14—C15	1.386 (4)
C3—C4	1.387 (3)	C14—H14	0.9500
C4—C5	1.382 (3)	C15—C16	1.391 (4)
C4—H4	0.9500	C15—H15	0.9500
C5—C6	1.398 (3)	C17—C18	1.513 (4)
C7—H7A	0.9800	C18—H18A	0.9800
C7—H7B	0.9800	C18—H18B	0.9800
C7—H7C	0.9800	C18—H18C	0.9800
			
C3—O1—C7	118.2 (2)	H8B—C8—H8C	109.5
C5—O2—C8	118.00 (19)	C10—C9—C1	125.3 (2)
C17—N1—C16	128.5 (2)	C10—C9—H9	117.3
C17—N1—H1	115.7	C1—C9—H9	117.3
C16—N1—H1	115.7	C9—C10—C11	125.8 (2)
C6—C1—C2	116.4 (2)	C9—C10—H10	117.1
C6—C1—C9	124.0 (2)	C11—C10—H10	117.1
C2—C1—C9	119.5 (2)	C12—C11—C16	118.6 (2)
C3—C2—C1	122.4 (2)	C12—C11—C10	122.6 (2)
C3—C2—Cl1	117.50 (19)	C16—C11—C10	118.8 (2)
C1—C2—Cl1	120.11 (19)	C13—C12—C11	120.9 (2)
O1—C3—C4	124.3 (2)	C13—C12—H12	119.6
O1—C3—C2	116.4 (2)	C11—C12—H12	119.6
C4—C3—C2	119.4 (2)	C14—C13—C12	120.1 (2)
C5—C4—C3	120.1 (2)	C14—C13—H13	119.9
C5—C4—H4	120.0	C12—C13—H13	119.9
C3—C4—H4	120.0	C13—C14—C15	120.3 (2)
O2—C5—C4	124.6 (2)	C13—C14—H14	119.8
O2—C5—C6	115.8 (2)	C15—C14—H14	119.8
C4—C5—C6	119.6 (2)	C14—C15—C16	119.8 (2)
C5—C6—C1	122.2 (2)	C14—C15—H15	120.1
C5—C6—Cl2	115.48 (18)	C16—C15—H15	120.1
C1—C6—Cl2	122.29 (19)	C15—C16—N1	122.6 (2)
O1—C7—H7A	109.5	C15—C16—C11	120.3 (2)
O1—C7—H7B	109.5	N1—C16—C11	117.0 (2)
H7A—C7—H7B	109.5	O3—C17—N1	123.4 (3)
O1—C7—H7C	109.5	O3—C17—C18	122.7 (3)
H7A—C7—H7C	109.5	N1—C17—C18	114.0 (2)
H7B—C7—H7C	109.5	C17—C18—H18A	109.5
O2—C8—H8A	109.5	C17—C18—H18B	109.5
O2—C8—H8B	109.5	H18A—C18—H18B	109.5
H8A—C8—H8B	109.5	C17—C18—H18C	109.5
O2—C8—H8C	109.5	H18A—C18—H18C	109.5
H8A—C8—H8C	109.5	H18B—C18—H18C	109.5
			
C6—C1—C2—C3	−0.7 (4)	C2—C1—C6—Cl2	176.94 (19)
C9—C1—C2—C3	174.7 (2)	C9—C1—C6—Cl2	1.8 (4)
C6—C1—C2—Cl1	179.43 (19)	C6—C1—C9—C10	−40.8 (4)
C9—C1—C2—Cl1	−5.2 (3)	C2—C1—C9—C10	144.2 (3)
C7—O1—C3—C4	−0.9 (4)	C1—C9—C10—C11	174.6 (2)
C7—O1—C3—C2	179.2 (2)	C9—C10—C11—C12	45.5 (4)
C1—C2—C3—O1	−178.1 (2)	C9—C10—C11—C16	−136.0 (3)
Cl1—C2—C3—O1	1.8 (3)	C16—C11—C12—C13	−0.6 (4)
C1—C2—C3—C4	2.0 (4)	C10—C11—C12—C13	177.9 (2)
Cl1—C2—C3—C4	−178.1 (2)	C11—C12—C13—C14	−0.9 (4)
O1—C3—C4—C5	177.6 (2)	C12—C13—C14—C15	1.5 (4)
C2—C3—C4—C5	−2.5 (4)	C13—C14—C15—C16	−0.5 (4)
C8—O2—C5—C4	−0.8 (4)	C14—C15—C16—N1	176.0 (2)
C8—O2—C5—C6	179.1 (2)	C14—C15—C16—C11	−1.1 (4)
C3—C4—C5—O2	−178.2 (2)	C17—N1—C16—C15	20.7 (4)
C3—C4—C5—C6	1.9 (4)	C17—N1—C16—C11	−162.1 (3)
O2—C5—C6—C1	179.5 (2)	C12—C11—C16—C15	1.6 (4)
C4—C5—C6—C1	−0.6 (4)	C10—C11—C16—C15	−177.0 (2)
O2—C5—C6—Cl2	2.3 (3)	C12—C11—C16—N1	−175.6 (2)
C4—C5—C6—Cl2	−177.7 (2)	C10—C11—C16—N1	5.8 (4)
C2—C1—C6—C5	0.0 (4)	C16—N1—C17—O3	0.3 (5)
C9—C1—C6—C5	−175.1 (2)	C16—N1—C17—C18	179.8 (2)
